# Solidification/stabilization of metallurgical tailings from the zinc process: environmental, microstructural, and mechanical aspects

**DOI:** 10.1007/s11356-026-37393-9

**Published:** 2026-01-24

**Authors:** Fernando Fante, Andrés Lotero, Hugo Carlos Scheuermann Filho, Giovani Jordi Bruschi, Maiki Mafessoli, Maria Alice Piovesan, Paulo Henrique Nogueira Metzker, Eduardo Pavan Korf, Nilo Cesar Consoli

**Affiliations:** 1 Graduate Program in Civil Engineering, UFRGS: Universidade Federal Do Rio Grande Do Sul, Porto Alegre, Rio Grande Do Sul 90035-190 Brazil; 2 Graduate Program in Environmental Science and Technology, UFFS: Universidade Federal da Fronteira Sul, Erechim, Rio Grande Do Sul 99700-970 Brazil; 3 Nexa Resources SA, Três Marias, MG 39205-000 Brazil

**Keywords:** Mine waste, Metallurgical tailings from the zinc process, Solidification/stabilization, Leaching tests, Laboratory tests

## Abstract

The management of metallurgical tailings from the zinc process (MTZP) involves a series of hazards due to the presence of heavy metals and sulfates in its composition. An alternative to overcome these difficulties is the use of the solidification/stabilization technique associated with the disposal in dry stacks. For this purpose, it was evaluated the environmental, microstructural, and mechanical properties of compacted metallurgical tailings from the zinc process stabilized with quicklime, Portland cement, and a mixture of both to reduce the potential contamination and increase the mechanical properties, under 7 and 28 days of curing. Analyses such as X-ray fluorescence spectrometry, X-ray diffraction, Fourier transform infrared spectroscopy, and scanning electron microscopy were conducted. Complementarily, mechanical response (unconfined strength and initial shear modulus) and batch leaching tests were executed to provide a complete understanding of treated material behavior. Stabilization with Portland cement reduced the leaching of heavy metals and was not effective in stabilizing the sulfates. The quicklime, due to the common ion effect, provided a reduction in the leaching of sulfates and some heavy metals and a better mechanical response compared to Portland cement. The use of both stabilizers produced the best combination of encapsulation of heavy metals, reduction in the leaching of sulfates, and enhancement of the mechanical properties.

## Introduction

Mine waste or mine tailings are generated during a beneficiation process in which the economically valuable component of the ore is separated from the non-valuable component (Piciullo et al. 2022). Due to the potential release of toxic pollutants that can threaten the environment and human health, these materials are generally managed in storage facilities or tailings dams (Rai et al. 2020; Chen et al. 2021, 2022). In this aspect, over the last few decades, attention has been drawn to the failures in tailings dams, which have caused several environmental disasters and human tragedies, with a total of 257 ruptures reported since 1915, with approximately 2650 fatalities and 250 million m^3^ of contaminated waste released into the environment (Piciullo et al. 2022). Two of these major catastrophes occurred in Brazil at the Fundão Dam in Mariana and the Córrego do Feijão Dam in Brumadinho (both in the province of Minas Gerais), which resulted in more than 278 deaths and 54 million m^3^ of tailings released (Do Carmo et al. 2017; Thompson et al. 2020; Silva Rotta et al. 2020). In this regard, dry stacking has been employed in Brazil as a possible solution for reducing the impacts of catastrophic dam failures, being an option for the de-characterization (decommissioning) process or new tailings/waste disposal (Mafessoli et al. 2023). More recently, cementing agents have been incorporated into the tailings/waste before the stacking, to provide an enhancement in the mechanical properties (Mafessoli et al. 2023; Ma et al. 2024; Cai et al. 2025; Khamseh et al. 2025).

Beyond the concerns about the safety of these structures, the release of hazardous contaminants through the leaching of the waste is another crucial aspect to consider (Huang et al. 2023; Wang et al. 2024). Leakage from ore beneficiation and leaching during rainfall events are examples of phenomena that result in the migration of heavy metals from mining areas to the environment (Peng et al. 2022; Wang et al. 2024). This contamination reaches watercourses, which become the main vehicle for the diffusion of the aforementioned pollutants (Desogus et al. 2013; Fei et al. 2017; Lin et al. 2018; Wang et al. 2024). Furthermore, when the sulfate is present in the waste, despite unbalancing the sulfur cycle, its accumulation in rivers may induce the release of toxic sulfides and, consequently, cause adverse impacts on the environment (Ghigliazza et al. 2000; Silva et al. 2002; Benatti et al. 2009). Therefore, the leaching of heavy metals and compounds such as sulfates, can inevitably bring great challenges to public health, as well as several risks to the environment (Bull and Fall 2020; Chen et al. 2021); Guimarães and Leão 2014; Liu et al. 2017; Ye et al. 2017).

The leaching of heavy metals in mining tailings/waste has been verified in several studies in recent years (Ghosh et al. 2011; Liu et al. 2017; Khoeurn et al. 2019; Chen et al. 2022). More specifically for MTZP, elevated levels of contaminants such as SO_4_, Fe, Mg, Ca, Cu, Pb, Ba, Zn, and S were detected (Othmani et al. 2013). Due to the presence of these pollutants and their leachability in the natural state, MTZP is often classified as hazardous solid waste (Luo et al. 2022).

An alternative to overcome this problem is the use of cementing agents (e.g., lime and Portland cement) to neutralize the contaminants through stabilization/solidification (García et al. 2004). Lime has been used alone or in mixtures in the treatment of soils contaminated with heavy metals (Akhter et al. 1990), expansive sulfated soils (Consoli et al. 2019), and gypsum soils (i.e., which have a high concentration of sulfates) (Aldaood et al. 2014a, c, b, 2021). The agent acts by increasing the pH of the medium, reducing the solubility of some heavy metals (Spence and Shi 2005), or retaining it through the products formed by pozzolanic reactions (Hossein 2000). Portland cement has been employed to stabilize contaminants and reduce their leaching, often associated with other agents such as fly ash, lime, bentonite, calcium aluminates, and blast furnace slag (Akhter et al. 1990; Hossein 2000; Spence and Shi 2005; Giergiczny and Król 2008; Barjoveanu et al. 2018; Zhang et al. 2021; Calgaro et al. 2021). The addition of cement creates a mechanical barrier within the matrix, decreasing permeability and preventing contaminants from leaching and being released into the environment (Hossein 2000; Spence and Shi 2005).

Several studies have addressed the stabilization of MTZP based on the use of cementing agents such as Portland cement and metakaolin (Zheng et al. 2018); Portland cement and bentonite (Chen et al. 2022); blast furnace slag and rice husk ash (Wang et al. 2022); Portland cement, lime, and fly ash (Erdem and Özverdi 2011); alkaline cement (Desogus et al. 2013; Wan et al. 2019; Chen et al. 2020); and even organic and inorganic chemical agents (Luo et al. 2022). However, in these studies, the MTZP had little or no sulfate in its composition. In this regard, there is a lack of research focused on the stabilization of metallurgical tailings from the zinc process (highly sulfated), for disposal in stacks. Thus, the present work aims to evaluate the environmental, microstructural, and mechanical properties of compacted metallurgical tailings from the zinc process stabilized with quicklime, Portland cement, and both to reduce the potential contamination and use in cemented piles.

## Materials

The waste used in this research was obtained from the hydrometallurgical process for metallic zinc production and was collected in the province of Minas Gerais, Brazil. It is important to note that while global zinc production predominantly relies on sulfide concentrates, whose processing generates significant jarosite residues, the material investigated herein derives from the beneficiation of silicate ores. The material presented a dominance of silt-sized particles (84.2%), in lesser proportions, fine sand (8.3%), clay (7.3%), and medium sand (0.2%). According to the Unified Soil Classification System (ASTM, 2017), the material was classified as a high-plasticity silt (MH). The specific gravity of solids was 2.64. The environmental classification conducted by the procedure of the NBR 10004 (ABNT, 2004a) indicated that the MTZP was classified as a waste class I—hazardous. In this aspect, the concentrations of As, Cd, and Pb exceeded the regulatory limit established by Annex F of NBR 10004 (ABNT, 2004a) as presented in Table 1. Based on the results of the X-ray fluorescence analysis (XRF), the predominant elements in the MTZP were sulfur (25.45%), silicon (17.79%), iron (15.59%), and calcium (14.10%), as can be seen in Table 2.

**Table 1:** Chemical composition of the leached extracts of the MTZP and mixtures (QL-MTZP, PC-MTZP, and QL-PC-MTZP) under 7 and 28 days of curing by the leaching test (mg/L)

Elements	MTZP	5QL-7d	5QL-28d	10PC-7d	10PC-28d	5QL-10PC-7d	5QL-10PC-28d	NBR	CONAMA 420	EPA	DL
Ag	0.00	0.00	0.00	0.01	0.00	0.04	0.00	5.00^a^	0.05	-	-
Al	0.00	0.00	0.00	0.00	0.00	0.00	0.00	-^a^	3.50	-	-
As	6.99	6.13	0.62	0.75	0.04	3.20	0.21	1.00^a^	0.01	0.01	0.01
Ba	0.00	0.00	0.49	0.00	0.00	0.73	0.00	70.00^a^	0.70	2.00	0.05
Cd	8.89	9.15	7.56	1.31	0.57	5.19	0.73	0.50^a^	0.005	0.005	0.0004
Cr	0.00	0.00	0.00	0.00	0.00	0.00	0.00	5.00^a^	0.05	0.10	0.001
Cu	0.11	1.99	2.14	0.00	0.00	0.00	0.00	-^a^	2.00	1.30	0.015
Fe	0.00	0.00	0.00	0.00	0.00	0.00	0.00	-^a^	2.45	-	-
Hg	0.00	0.00	0.00	0.00	0.00	0.00	0.00	0.10^a^	0.001	0.002	0.00005
Mn	33.20	33.00	35.50	25.35	9.87	36.70	7.88	-^a^	0.40	-	-
Na	0.00	0.00	0.00	0.00	0.00	0.00	0.00	-^a^	-	-	-
Pb	7.75	27.15	62.13	3.17	1.15	3.10	0.50	1.00^a^	0.01	0.015	0.015
Se	0.18	0.57	0.44	0.00	0.00	0.00	0.00	1.00^a^	0.01	0.05	-
Zn	439.00	589.94	471.19	288.44	145.94	342.94	99.74	-^a^	1.05	-	0.065
pH	5.12	4.74	4.78	5.12	5.03	6.30	6.08	-^a^	-	6.50–8.50	-

^a^NBR 10004–Annex F

**Table 2:** X-ray fluorescence analysis of the materials (MTZP, quicklime, and Portland cement) and the mixtures (QL-MTZP, PC-MTZP, and QL-PC-MTZP)

Elements	MTZP	QL	PC	5QL-7d	5QL-28d	10PC-7d	10PC-28d	5QL-10PC-7d	5QL-10PC-28d
Si	17.79	0.22	27.30	16.15	17.11	19.44	18.12	17.25	17.33
Al	1.09	0.04	7.36	1.00	0.98	1.59	1.45	1.31	1.33
Ti	n.d	n.d	n.d	n.d	n.d	0.01	0.01	0.01	0.01
Fe	15.59	0.11	1.67	14.87	14.56	14.31	14.20	14.15	13.25
Mn	0.18	n.d	0.43	0.19	0.21	0.21	0.20	0.21	0.22
Mg	1.84	2.32	6.19	1.87	2.04	2.89	2.18	2.39	2.60
Ca	14.10	82.45	49.11	16.74	16.83	15.96	17.11	19.40	19.34
Na	1.01	n.d	0.21	0.88	0.94	1.04	0.86	0.90	0.91
K	0.18	0.01	0.32	0.18	0.18	0.22	0.22	0.21	0.21
P	0.08	n.d	0.19	0.08	0.07	0.08	0.08	0.08	0.07
Cl	n.d	n.d	n.d	n.d	n.d	0.01	0.03	0.00	0.01
Cu	0.05	n.d	n.d	n.d	n.d	n.d	n.d	n.d	n.d
Ni	n.d	n.d	n.d	n.d	n.d	n.d	n.d	n.d	n.d
Co	n.d	n.d	n.d	n.d	n.d	n.d	n.d	n.d	n.d
Cd	0.07	n.d	n.d	0.07	0.08	0.07	0.07	0.07	0.07
Pb	2.53	n.d	n.d	2.43	2.40	2.30	2.34	2.31	2.19
Zn	2.21	n.d	0.03	2.23	2.40	2.11	2.29	2.13	2.22
Ba	0.19	n.d	n.d	0.19	0.18	0.19	0.19	0.19	0.18
S	25.45	2.25	3.13	25.40	26.48	23.73	25.45	24.50	24.87
LOI	17.64	12.60	3.35	17.79	15.50	15.78	15.17	14.86	15.15

*n.d.* not detected, *LOI* loss of ignition

**Fig. 1:**
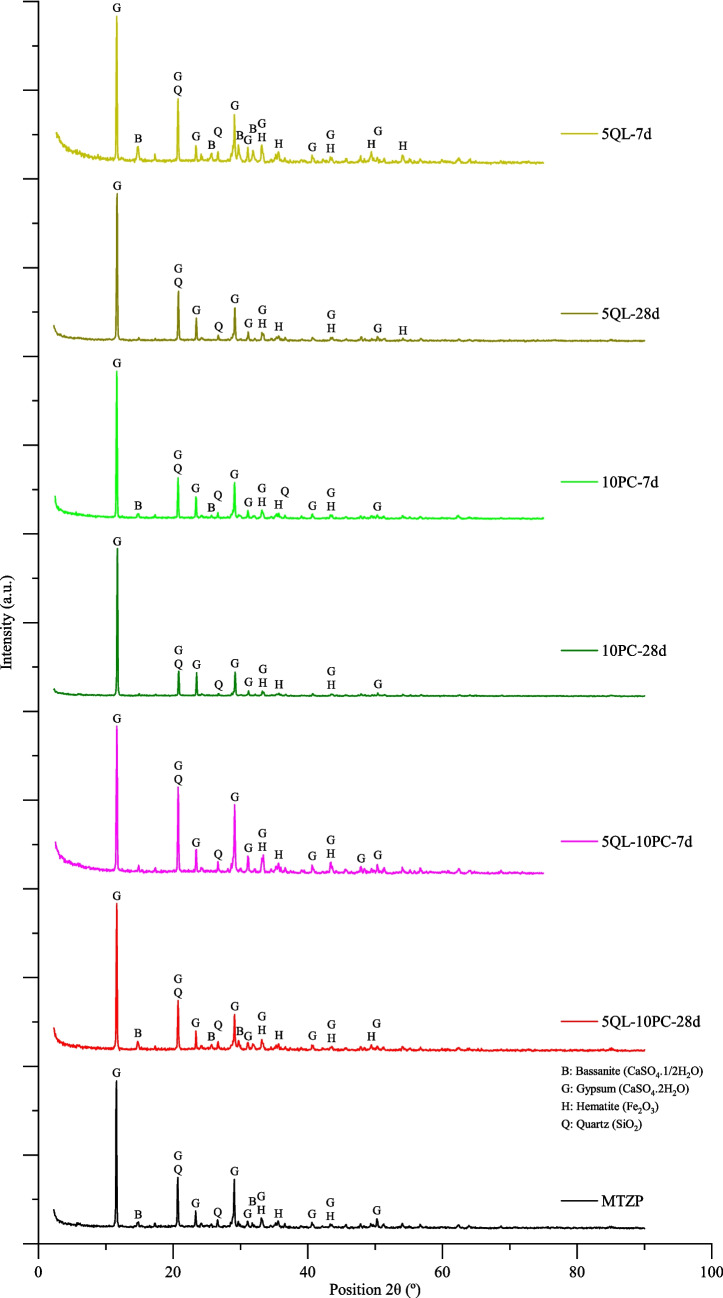
XRD for the MTZP and the mixtures (QL-MTZP, PC-MTZP, and QL-PC-MTZP)

**Fig. 2:**
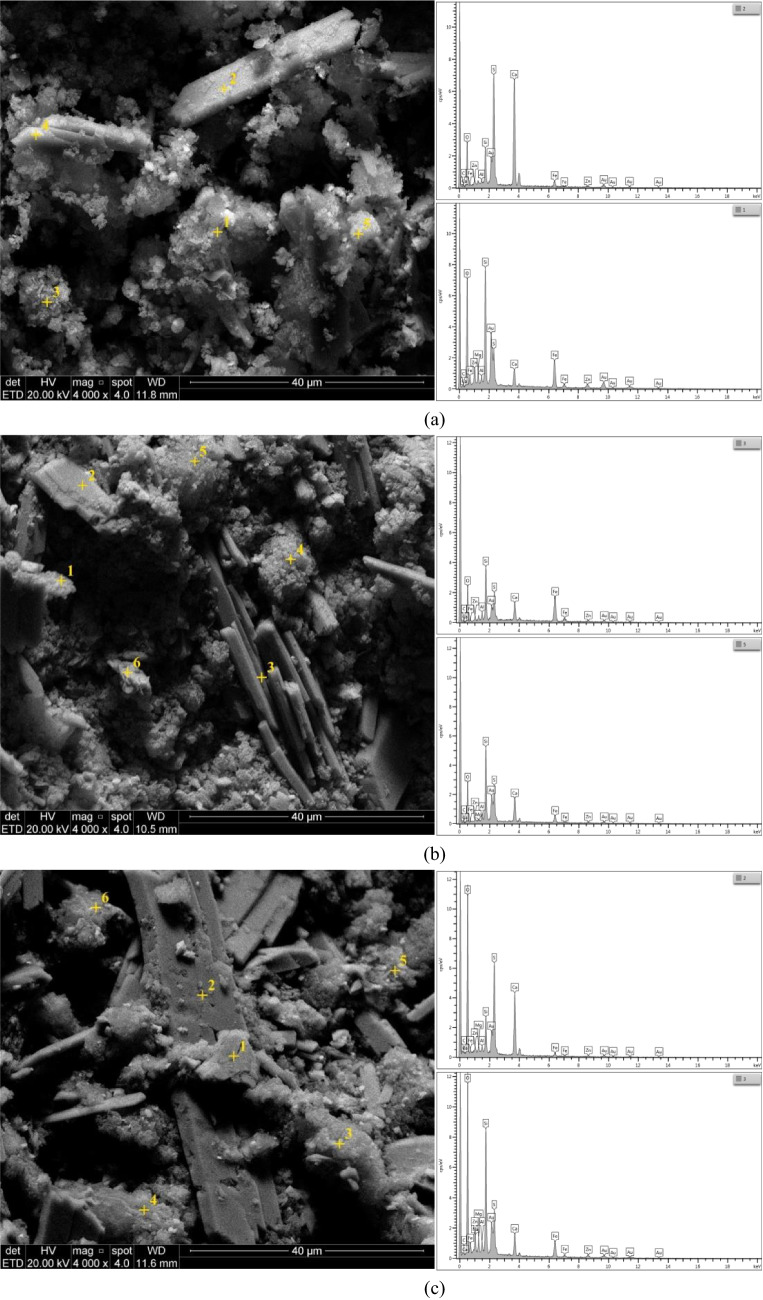
SEM–EDS, 4.000 times magnification: **a** MTZP, **b** 5QL-28d, and **c** 10PC-28d

From the X-ray diffraction (XRD) analysis (Fig. 1), it was detected some crystalline structures such as bassanite (CaSO_4_·1/2H_2_O), gypsum (CaSO_4_·2H_2_O), hematite (Fe_2_O), and quartz (SiO_2_) in the MTZP, corroborated by the detection of S, Si, Fe, and Ca in the XRF evaluation. Complementarily, from the microstructural analyses (SEM–EDS) performed on MTZP (Fig. 2), it can be observed elongated prismatic structures resembling “sticks” and plate-shaped particles suggesting a sulfate morphology (Seewoo et al. 2004; Chen et al. 2021). This finding was supported by the EDS analysis conducted at point 2 (Fig. 2a), which detected important amounts of Si, Ca, and O. Small clusters of crystals and the existence of iron, oxygen, and silicon identified in the EDS (point 1) indicated the presence of hematite and quartz.

**Fig. 3:**
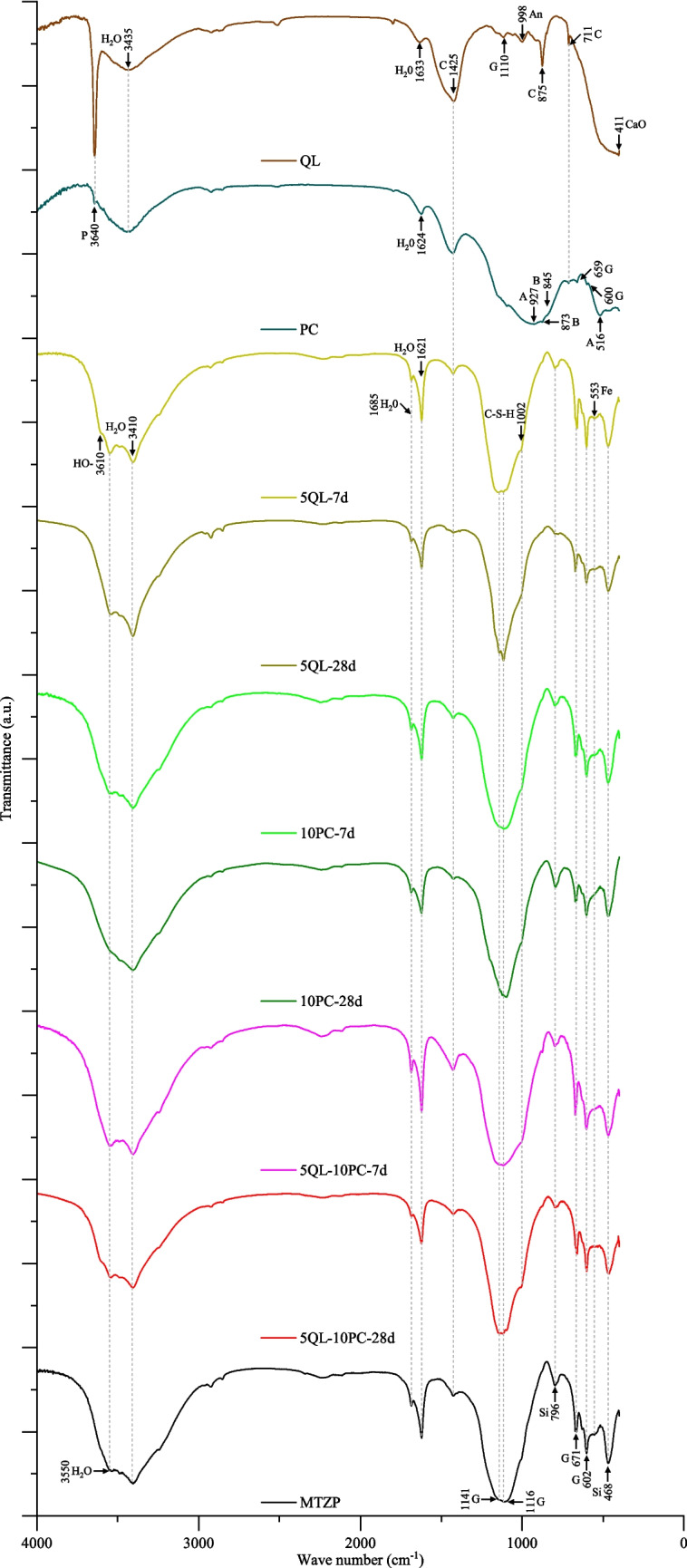
FT-IR spectra for the materials (MTZP, quicklime, and Portland cement), and the mixtures (QL-MTZP, PC-MTZP, and QL-PC-MTZP). Notes: water (H_2_O), calcite (C), gypsum (G), silicates (Si), iron minerals (Fe), portlandite (P), alite (A), belite (B), anhydrite (An), and calcium oxide (CaO)

The stabilization/solidification of the metallurgical tailings from the zinc process was conducted by the use of quicklime and blast furnace slag Portland cement (type III RS). The XRF carried out in these materials (Table 2) evidenced the presence of calcium, silicon, magnesium, aluminum, and sulfur as the main elements of PC and calcium, magnesium, and sulfur for the QL. The Fourier transform infrared spectroscopy (FT-IR) analysis performed in QL and PC (Fig. 3) indicated a band located at 3640 cm^−1^ that was associated with the stretching vibrations of O–H bonds in portlandite (Zaki et al. 2006; Domínguez et al. 2008; García Lodeiro et al. 2009; Taddei et al. 2009; Jose et al. 2020). The band at 3435 cm^−1^ was attributed to stretching vibrations in O–H bonds and the region between 1624 and 1633 cm^−1^ to bending vibrations in H–O-H, both from water molecules present in the sulfate (Zaki et al. 2006; Król et al. 2016; Bulatović et al. 2017; Moukannaa et al. 2018; Khan et al. 2019; Jose et al. 2020).

The broad band centered at 1425 cm^−1^ and peaks at 875 cm^−1^ and 711 cm^−1^ were characteristic of the stretching of the C-O bonds, due to the formation of calcite through carbonation processes (Yousuf et al. 1993; Lane and Christensen 1997; Reig 2002; Zaki et al. 2006; García Lodeiro et al. 2009; Ji et al. 2009; Pacheco-Torgal et al. 2015; Król et al. 2016; Khan et al. 2019; Saedi et al. 2023). The peak at 1110 cm^−1^ corresponds to the vibration of sulfates (SO_4_) while the absorption bands at 690 cm^−1^, 659 cm^−1^, and 600 cm^−1^ advocate the presence of gypsum or ettringite minerals (Taddei et al. 2009; Bulatović et al. 2017; Khan et al. 2019). The specific phases of the clinker recognized at 927 cm^−1^, 873 cm^−1^, 845 cm^−1^, and 516 cm^−1^ were attributed to silicate ions as alite or tricalcium silicate (C_3_S), and belite or dicalcium silicate (C_2_S) (Yousuf et al. 1993; Domínguez et al. 2008; Taddei et al. 2009; Saedi et al. 2023). On the other hand, intense broadband at 400 cm^−1^ was verified in quicklime, which represents the Ca-O bond (Zaki et al. 2006), while at 998 cm^−1^ it implies the existence of anhydrite (Sarma et al. 1998; Taddei et al. 2009).

## Methods

### Experimental design

The experimental design was performed with three different mixtures QL-MTZP, PC-MTZP, and QL-PC-MTZP, to elucidate the behavior of these distinguished stabilizers. The stabilizer content was defined based on the literature as 5% for quicklime (Ingles and Metcalf 1973; Mitchell 1981; U.S. U.S. Army Corps of Engineers 1994; Consoli et al. 2019) and 10% for Portland cement (Mitchell 1981; Consoli et al. 2010). These values were consistent with previous studies on the stabilization of lead–zinc mine tailings (Desogus et al. 2013; Chen et al. 2022) and hydrometallurgical zinc waste or jarosite (Mymrin and Vazquez Vaamonde 1999; Gupta and Prasad 2018). Two curing times (7 and 28 days) were utilized for a better understanding of chemical, mineralogic, microstructural, and leaching behavior over time. The moisture content and the dry unit weight were defined based on the results of compaction tests conducted in MTZP under normal effort (*w*_opt_ = 64% and γ_d_ = 9.20 kN/m fixed for all specimens). Since temperature effects were beyond the scope of this study, it was consequently maintained at 23 °C. Finally, to indicate the mechanical behavior of the mixtures, initial shear modulus and unconfined compression tests were conducted for 7 and 28 days of curing.

### Molding and curing

Due to the presence of gypsum, a specific procedure was followed during the material preparation as stated by D2216 (ASTM, 2019), which involves drying the MTZP at 60 ± 2 °C for 48 h. Afterward, the components of the mixture (MTZP, quicklime, Portland cement, and destilled water) were weighed, and the dry constituents were mixed until a homogeneous blend was achieved. The destilled water was then gradually added to the dry blend and mixed until a homogeneous paste was obtained. After that, the mass of each layer was weighed and placed into plastic containers to prevent humidity loss. The specimen was statically compacted in three layers following the undercompaction method (Ladd 1979), into a lubricated, three-part cylindrical mold (50 mm in diameter and 100 mm in height). Immediately after molding, the specimen was extracted, and its weight and dimensions (diameter and height) were measured with accuracies of 0.01 g and 0.1 mm, respectively. Finally, the sample was placed in airtight bags and cured in the humidity room at 23 ± 2 °C and relative humidity of 95%.

### Leaching

The leaching tests were conducted following the procedure presented in the Brazilian standard NBR 10005 (ABNT 2004b), the solubility tests by the NBR 10006 (ABNT, 2004c), and the solid waste characterization by the NBR 10004 (ABNT 2004a). For the leaching tests, after reaching the curing time, the specimens were crushed, sieved through a mesh with an opening of 9.5 mm, and exposed to an acetic acid solution with a pH of ~ 2.8 through a solid/liquid ratio of 1:20. Finally, the mixture was agitated in a rotary shaker at a fixed rotation of 30 rpm, for 18 ± 2 h and 23 ± 2 °C. Subsequently, the leachate was kept in a temperature-controlled environment (< 4 °C) for further filtration, pH, and metal determination. A membrane filter with an opening of 0.45 µm was used for filtering the leachate. The metal concentrations were determined by inductively coupled plasma optical emission spectrometry (ICP-OES), executed in a Shimadzu ICP Emission Spectrometer (model ICPE-9800). The standard curves were prepared into a mono-element solution in which elements were diluted in nitric acid HNO_3_.

For the solubility tests, the specimens after achieving the curing time were crushed, sieved through a mesh with an opening of 9.5 mm, and exposed to distilled water through a solid/liquid ratio of 1:4. Finally, the mixture was agitated in a rotary shaker at a low rotation for 5 min. Subsequently, the extract was kept for 7 days in a temperature-controlled environment (< 25 °C) for further filtration, pH, and sulfate determination. A membrane filter with an opening of 0.45 µm was employed for filtering the extract. The turbidimetric analysis (Sheen et al. 1935) through UV–visible spectrometry was employed to evaluate the sulfate concentration. The apparatus consisted of a Kasuaki Spectrophotometer (model IL-592), operating in the UV–visible light range of 195 to 1020 nm, featuring a ± 2 nm bandwidth, single-beam configuration, and silicon photodiode detector. The tests were carried out using a 420-nm wavelength, quartz cuvettes, in duplicate, and following the procedures of APHA, AWWA, and WEF (2017). The pH of the extracts was measured by Hanna pH meter (model HI 2221), glass body, and Ag/AgCl electrode. The element’s concentration was compared with the EPA (USEPA 2009), the Dutch List (VROM 2000), CONAMA 420 (CONAMA 2009), and NBR 10004 (ABNT 2004a).

### Microstructural behavior

After the curing time, treated MTZP specimens used to investigate microstructural behavior were crushed, dried, and promptly submitted for analysis. For the cases where a powder material was used in the tests, an additional step of sieving through a #325 mesh was employed. The raw materials (QL, PC, and MTZP) were prepared in a simplified manner, where they were only dried and sieved before analysis. The elemental composition was determined by XRF spectrometry analysis carried out on an RIX 2000 (Rigaku) spectrometer equipped with a Rh tube and refrigerated anode, with tabulated rock pattern calibration curve developed by Geostandars and the sample preparation by pressed powder method. The mineralogical composition was verified by XRD analysis, conducted with a D-5000 (θ−2θ) (Siemens) diffractometer, equipped with a fixed Cu anode tube (λ = 1.5406 Å), operating at 40 kV and 25 mA in the primary beam and curved graphite monochromator in the secondary beam. The samples were analyzed in the angular range of 2.3° to 90° 2θ in a step of 0.05°/1 s. The chemical bonds analysis was conducted through FT-IR, employing *Spectrum* 1000 (PerkinElmer) FT-IR spectrometer, operating within the range of 4000–400 cm^−1^ and at a resolution of 4 cm^−1^. Finally, the microstructural evaluation was executed by scanning electron microscopy (SEM), performed in a JSM-6610LV (Jeol) microscope, using an electron beam of 20 kV, and gold-coated samples and with magnifications of 4000 times. The qualitative identification of the elements in the microstructural analysis was due to energy-dispersive X-ray spectroscopy (EDS) through a Nano X Flash Detector 5030 (Brucker) energy-dispersive spectrometer coupled to the microscope.

### Mechanical response

Assuming the material is elastic, homogeneous, and isotropic, the initial shear modulus can be obtained from elasticity theory relating the shear wave velocity (*V*_s_) and the apparent density (ρ), as presented in Eq. 1 (Mitchell and Soga 2005):

1 $${G}_{0}=\rho \bullet {V}_{s}^{2}$$

The shear wave velocities were determined by an electronic pulse device that emits shear waves at a constant frequency of 250 kHz. Transducers were attached at the top and bottom of the specimens, employing coupling gel.

The unconfined compression tests were executed following the D2166 (ASTM 2016), through an automatic loading press, operating at a displacement rate of 1.14 mm/min. A 5-kN load cell was attached to the loading press to measure the maximum applied load. Before the tests, the specimens were submerged in water for 24 h to reduce the suction effects (Mafessoli et al. 2023).

## Results and discussion

### Leaching tests

Table 1 presents the concentration of the metals in the leached extracts of MTZP, QL-MTZP, PC-MTZP, and QL-PC-MTZP mixtures from the batch leaching tests. The concentrations of silver in the MTZP, as well as for the QL-MTZP, PC-MTZP, and QL-PC-MTZP extracts, were below the limits specified by the CONAMA 420 (CONAMA 2009) and NBR 10004 (ABNT 2004a). A small concentration of Ag was detected in MTZP treated with PC, probably as an impurity within the cement, since this metal was not verified in MTZP and in treatments using only QL. Similarly, aluminum was not identified in the MTZP extract and, consequently, was below the established limits of CONAMA 420 (CONAMA, 2009). Unlike ordinary Portland cement which contains a significant amount of tricalcium aluminate (C_3_A), sulfate-resistant cements have low quantities of this compound (Bye 2011). This fact, associated with the non-leaching of aluminum in the mixtures, implies that the small amount present in the stabilizers was consumed in pozzolanic reactions to form calcium aluminate hydrate (C-A-H) gel, a reaction product of Portland cement (Bergado et al. 1996; Taylor 1997) and quicklime (Bergado et al. 1996).

The concentrations of As in MTZP extract were above all the regulatory limits evaluated. After the incorporation of quicklime, Portland cement, and both, the concentrations were reduced at 7 days of curing and again at 28 days of curing. In this aspect, just the 28 days of curing QL-MTZP, PC-MTZP, and QL-PC-MTZP mixtures satisfy the limits of NBR 10004 (ABNT, 2004a). For the studied conditions, the treatment with Portland cement presented a greater effectiveness in the immobilization of As when compared with quicklime. The efficacy of the treatment of As with Portland cement was also verified by other studies (Akhter et al. 1990; Pereira dos Santos et al. 2024). Barium was not detected in the MTZP extract and, consequently, attained all regulatory limits evaluated. It should be noted that when quicklime was added to the MTZP, an increase in metal concentrations in the extract was observed, due to the presence variable of Ba in limestones and clays, which are raw materials for clinker and lime (Vollpracht and Brameshuber 2016; Ferrazzo et al. 2023). From this perspective, only the treatment with Portland cement meets the minimum normative requirements.

Conversely, the Cd concentration in the MTZP extract was above the evaluated regulations. The inclusion of quicklime produced a slight decrease in the concentrations of cadmium, credited to the incorporation of the Cd ion into the portlandite Ca(OH)_2_ molecule. The consequence is the replacement of Ca, and the formation of a double compound CdCa(OH)_4_ (Park 2000), or precipitation as Cd(OH)_2_ (Cartledge et al. 1990). The incorporation of PC decreased the concentration of Cd by encapsulating it within the cemented matrix, specifically by the C-S–H/C-A-S–H gels types (Ferrazzo et al. 2023). For the studied treatments, the PC-MTZP mixture provided greater efficiency in the reduction of leachability of Cd; however, it was not possible to fully meet the regulatory limits of NBR 10004 (ABNT 2004a), CONAMA 420 (CONAMA 2009), EPA (USEPA 2009), and DL (VROM 2000). The presence of Cr, Fe, Hg, and Na was not observed in the MTZP extract, as well as it was not identified after the treatments with quicklime, Portland cement, and both.

The concentration of copper in the extract of MTZP was above the regulatory limits of the Dutch List (VROM 2000). The leaching of the Cu was controlled by the dissolution/precipitation of Cu(OH)_2_ and CuO, with CuO being an amphoteric oxide (Özkök et al. 2013; Mahedi et al. 2019). This aspect may explain the increase in copper leaching as the pH moves away from neutral, especially for the QL-MTZP. As reported in the literature, Portland cement may encapsulate the Cu in the cement matrix (Pereira dos Santos et al. 2024), which explains the zero value for the treatment with Portland cement. Therefore, the PC-MTZP and QL-PC-MTZP treatments attained the regulatory standards of NBR 10004 (ABNT, 2004a), CONAMA 420 (CONAMA 2009), EPA (USEPA 2009), and DL (VROM 2000). For the Mn, it was noted that its concentration in the extract of MTZP was considerably above the regulatory limit of CONAMA 420 (CONAMA, 2009). The inclusion of QL practically did not change the metal levels, unlike the incorporation of PC which provided a reduction in manganese leaching. It occurred due to the precipitation of Mn in the form of manganese hydroxide Mn(OH)_2_ (Cetin and Aydilek 2013), providing its encapsulation (Wang et al. 2020, 2023; Han et al. 2023). Despite the reduction in the metal leaching with the PC-MTZP treatment, the CONAMA 420 (CONAMA 2009) standard was not satisfied.

In the case of lead, its concentration in the MTZP was above all the regulatory limits evaluated. The treatment with PC provided a reduction in the leaching due to the replacement of divalent cations as Ca^2+^ by the Pb^2+^ in cement hydrate phases (Spence 1993; Gougar et al. 1996). Moreover, it was reported that Pb presented lower solubility at the range of pH 7.5–10 (Shnorhokian 1996), which explained the increase in leaching observed in the QL-MTZP treatment. Thus, although there was a decrease in the metal concentrations, the regulatory limits were not attained. For selenium, the concentration in the MTZP extract was only below the NBR 10004 (ABNT 2004a) limit. The inclusion of Portland cement provided a reduction of the levels of Se, due to its encapsulation in the cemented matrix (Pereira dos Santos et al. 2024), and as a consequence, attaining the regulatory limits of NBR 10004 (ABNT, 2004a), CONAMA 420 (CONAMA 2009), and EPA (USEPA 2009). The concentration of zinc in the MTZP extract was above the regulatory limits, as was also observed by Sethurajan et al. (2016) in the extract from metallurgical waste from the zinc plant. The treatments with Portland cement decreased the metal leaching for the studied curing times, due to the encapsulation of the contaminant in the cement matrix (Malviya and Chaudhary 2006). Although a reduction in the leaching was noted, the regulatory standards of CONAMA 420 (CONAMA 2009) and DL (VROM 2000) were not met.

Table 3 shows the concentration of sulfate in the solubilized extracts of MTZP, QL-MTZP, PC-MTZP, and QL-PC-MTZP mixtures from the batch tests. It can be noted that a high concentration of sulfate was detected in the MTZP extract, which was above the regulatory limits of NBR 10004 (ABNT 2004a) and EPA (USEPA 2009). A considerable decrease in leaching was observed when the MTZP was stabilized with quicklime. In this regard, the CaO reacts with H_2_O, releasing heat and forming portlandite Ca(OH)_2_ (Spence 1993; Bergado et al. 1996; Pacheco-Torgal et al. 2015; Ineich et al. 2017).

**Table 3:** Chemical composition of the solubilized extracts of the MTZP and mixtures (QL-MTZP, PC-MTZP, and QL-PC-MTZP) by the leaching test (mg/L)

Elements	MTZP	5QL-7d	5QL-28d	10PC-7d	10PC-28d	5QL-10PC-7d	5QL-10PC-28d	NBR	CONAMA 420	EPA	DL
SO_4_	5191.97	2784.1	2658.69	6195.25	5668.53	2733.94	2683.77	250.00^a^	-	250.00	-
pH	3.61	6.43	7.01	4.61	6.11	6.54	6.42				

^a^NBR 10004–Annex G

The portlandite dissociates when it enters in contact with soil water, generating Ca^2+^ and 2(HO)^−^ ions and increasing the pH of the system (Bergado et al. 1996; Pacheco-Torgal et al. 2015). Calcium ions combine with sulfate ions and end up forming gypsum (Bulatović et al. 2017). Due to the excess of calcium ions common to calcium sulfate ions (i.e., common ion effect), there is a reduction in solubility due to precipitation in the form of gypsum (Benatti et al. 2009; Deng et al. 2013; Roy and Bhattacharya 2015; Ineich et al. 2017; Nariyan et al. 2018). In other words, a solid precipitate/crystal is formed when a solute exceeds its solubility in an aqueous solution. Therefore, the precipitation process in the form of gypsum is the main way of controlling sulfate dissolution (McGregor et al. 1998; Doye 2004; Astrup et al. 2006; Romero et al. 2007; Nehdi and Tariq 2007). On the other hand, when only Portland cement was added to the system, there was not sufficient Ca^2+^ to produce the common ion effect which precipitated the gypsum. Therefore, the Portland cement did not provide a reduction in sulfate leaching. This aspect was supported by the QL-PC-MTZP results, in which it can be seen that the decrease in sulfate leaching is of the same order of magnitude as that of the treatment QL-MTZP.

The batch leaching tests revealed that Portland cement was generally more effective than quicklime in reducing the leachability of most metals (As, Cd, Cu, Mn, Pb, Se, and Zn), although not all final concentrations achieved regulatory standards. In this aspect, the PC stabilizes the heavy metals primarily through the encapsulation within the C-S–H gel (Chen et al. 2009). This is also corroborated by the microstructural analysis, which confirmed the formation of this gel. On the other hand, quicklime showed limited or even adverse effects on metal leaching, attributed to pH changes and potential impurities. Nevertheless, quicklime stabilizes sulfates through the gypsum precipitation driven by the common ion effect. The combined use of Portland cement and quicklime provides a synergistic effect through the encapsulation of heavy metals within the C-S–H gel and the stabilization of the sulfate due to the common-ion effect and gypsum formation.

### Microstructural behavior and mechanical response

Table 2 presents the XRF analysis performed in the QL-MTZP, PC-MTZP, and QL-PC-MTZP mixtures at distinct curing times. According to the results, the chemical composition of QL-MTZP, PC-MTZP, and QL-PC-MTZP mixtures was similar to the MTZP, with high concentrations of sulfur, calcium, silicon, and iron. The inclusion of quicklime and Portland cement provoked an increase in the calcium content because of its presence in the composition of the stabilizers. The low sulfur content in quicklime and Portland cement resulted in little variations in QL-MTZP, PC-MTZP, and PC-MTZP mixtures. Remarkably, heavy metals such as barium, zinc, lead, and cadmium were observed in the mixtures; however, their presence was attributed to MTZP.

XRD analysis performed in the MTZP and the mixtures QL-MTZP, PC-MTZP, and PC-MTZP under different curing times are exhibited in Fig. 1. Some crystalline structures such as bassanite (CaSO_4_·1/2H_2_O), gypsum (CaSO_4_·2H_2_O), hematite (Fe_2_O), and quartz (SiO_2_) were identified in the QL-MTZP, PC-MTZP, and QL-PC-MTZP mixtures in all curing times, with their presence being credited to the MTZP. This aspect was in agreement with the data obtained in the XRF analysis, where a relevant amount of calcium, sulfur, iron, and silicon were detected (see Table 2). Some peaks from the MTZP were maintained in the stabilized material, demonstrating that these minerals were not dissolved and remained within their original structures throughout the studied curing times. Peaks like 14.75° and 25.72° attributed to the bassanite mineral disappear over time, indicating that phase was consumed during the cementitious reactions.

FT-IR analysis executed in the MTZP and for the mixtures QL-MTZP, PC-MTZP, and PC-MTZP under different curing times is presented in Fig. 3. It is important to mention that some stretching bands in the mixtures were also identified in the MTZP, which implies that they are derived from the same material. The stretching bands at 3550, 3410, 1685, 1621, 1141, 1116, 671, and 602 cm^−1^ were credited to sulfate (Estep et al. 1968; Yousuf et al. 1993; Taddei et al. 2009; Kamel et al. 2015; Hu et al. 2017; Mechri et al. 2017; Bulatović et al. 2017; Ye et al. 2017; Khan et al. 2019; Jose et al. 2020). The stretching bands at 3610, 3550, and 3410 cm^−1^ were associated with the vibrations of the O–H bonds, whereas the bands located at 1685 and 1621 cm^−1^ were characteristic of the bend vibrations of H–O-H bonds. In both cases, the vibrations were attributed to the water molecules within the sulfate structure (Yu et al. 1999; Zaki et al. 2006; Pacheco-Torgal et al. 2015; Król et al. 2016; Moukannaa et al. 2018; Jose et al. 2020), or the manifestation of the hemihydrate form of the sulfate (bassanite), more specifically at 3610 and 3550 cm^−1^ (Estep et al. 1968; Mechri et al. 2017). The vibrations located at 3610, 3550, 3410, 1685, and 1621 cm^−1^ reduced their intensities during the curing. It can be attributed to the dehydration of bassanite (consumption of H_2_O) during the hydration reactions. This aspect followed the XRD results, which also demonstrated a decrease in the peak intensity of bassanite over the curing period. The vibration at 1425 cm^−1^ corresponded to the stretching vibration of the C-O bond, which suggested the formation of calcite (CaCO_3_) through the carbonation process (Yu et al. 1999; García Lodeiro et al. 2009; Pacheco-Torgal et al. 2015; Król et al. 2016; Saedi et al. 2023). Through the setting time, there was a decrease in the intensity of the band, caused by the reduction of the calcite due to hydration reactions (Jose et al. 2020). Additionally, low-intensity peaks were identified at 553 cm⁻^1^ which referred to the stretching of Fe–O bonds characteristic of iron oxides (Fischer 1975; Sidhu 1988; Vempati 1990; Hofmeister et al. 2001).

Moreover, the identification of a shoulder at the wavenumber 1002 cm⁻^1^ in all mixtures and curing times suggested the formation of cementing compounds. This finding was supported by the literature, where the wavenumbers 1002 and 1090 cm⁻^1^ were frequently associated with stretching vibrations of silicates (Si–O or Si–O-Si) observed in the C-S–H gel (Yu et al. 1999; Criado et al. 2007; Ahmari and Zhang 2013; Sáez del Bosque et al. 2014; Yaseri et al. 2019; Saedi et al. 2023). Throughout the curing time, variations in the relative intensities at 796 and 468 cm^−1^ were observed. Since these vibrations were credited to the silicate groups (Si–O and Si–O-Si), it may indicate a probable reorganization and formation of C-S–H (Zhang et al. 2016; Jose et al. 2020).

**Fig. 4:**
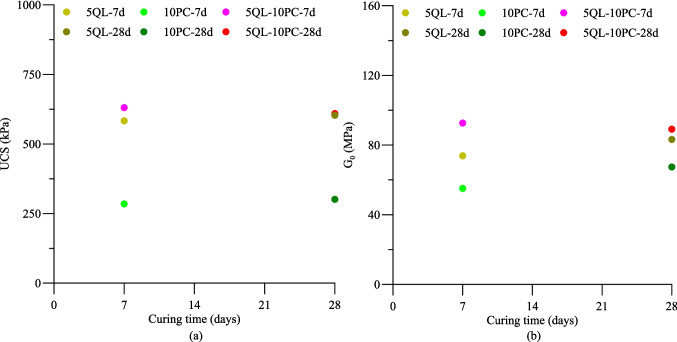
Mechanical behavior of QL-MTZP, PC-MTZP, and QL-PC-MTZP mixtures under 7 and 28 days of curing: **a** unconfined compression strength, and **b** initial shear modulus

This aspect was manifested in the mechanical response through the unconfined compressive strength (UCS) and initial shear modulus (G_0_), as shown in Fig. 4. An increase in compressive strength and the initial shear modulus when compared with the MTZP was observed. For the three tested mixtures, the UCS exceeded the 200 kPa threshold suggested by Daniel and Wu (1993) for liners and verified by Ribeiro et al. (2026) and Viana da Fonseca et al. (2024) for application on cemented berms. In this regard, the improvement in mechanical properties is particularly advantageous for low-height tailings piles (< 30 m) or in cemented berms, preventing local slope failures.

The enhancement in strength and stiffness can be attributed to the formation of cementitious materials, specifically calcium silicate hydrate (C-S–H) (Taylor 1997; Prusinski and Bhattacharja 1999), as demonstrated in the FT-IR analysis. Likewise, the UCS and G_0_ essentially do not vary with the curing time for all tested mixtures. The findings indicated that most of the strength and stiffness gains occurred at early ages, with the QL-PC-MTZP treatment exhibiting a more favorable response. Nevertheless, the difference in the mechanical response between the QL-PC-MTZP and QL-MTZP mixtures is practically negligible and may be associated with the content of Ca in the system, as will be addressed below.

There are two different types of reactions that can be observed when dealing with a lime-stabilized material: modification (including hydration, cation exchange, and agglomeration/flocculation) and stabilization (pozzolanic reactions and carbonation) (Rogers and Glendinning 2000; Nicholson 2015; Aldaood et al. 2021). The increase in strength of the stabilized material was developed to the pozzolanic reactions that led to the formation of reaction products such as calcium silicate hydrates (C-S–H) and calcium aluminate hydrates (C-A-H) (Bergado et al. 1996; Aldaood et al. 2021). Such products depend on a source of silica and alumina, which can be found in the clay fraction of the soil (Bergado et al. 1996; Pacheco-Torgal et al. 2015). As mentioned above, the quicklime reacts with water releasing heat and forming portlandite (Spence 1993; Bergado et al. 1996; Pacheco-Torgal et al. 2015; Ineich et al. 2017). The portlandite dissociates into Ca^2+^ and 2(HO)^−^ ions when exposed to water, raising the pH, which, in turn, leads to the dissolution of silica from the clay minerals (Bergado et al. 1996; Pacheco-Torgal et al. 2015). The calcium from the quicklime (portlandite) combined with the dissolved silica and formed calcium silicate (Transportation Research Board 1987).

The consumption of portlandite due to the formation of calcium silicate is evidenced in the FT-IR analysis. A close examination of the quicklime spectrum in Fig. 3 revealed a stretching vibration at 3640 cm^−1^ that can be attributed to the O–H bonds of portlandite (Zaki et al. 2006; Domínguez et al. 2008; García Lodeiro et al. 2009; Taddei et al. 2009; Jose et al. 2020). Despite its presence in the quicklime, the absence of this band in the QL-MTZP or QL-PC-MTZP mixtures indicated that it was consumed through the pozzolanic reactions with the minerals in the tailings (Spence and Shi 2005). As part of the available calcium was previously consumed during the precipitation of sulfate (SO₄) as calcium sulfate (CaSO₄) due to the common ion effect (Table 3), only the remaining Ca formed the reaction products. Therefore, since sulfate precipitation precedes pozzolanic reactions, the products can encapsulate the calcium sulfate within the cementitious matrix, as can be seen in Fig. 2b for QL-MTZP under 28 days of curing. The presence of sulfates was supported by the verification of an elongated, rod-shaped particle morphology (Seewoo et al. 2004; Chen et al. 2021) associated with the existence of calcium, sulfur, and oxygen which was observed in the EDS analysis at point 3. The rod-shaped particles were surrounded by a spongy morphology (formed by Ca, Si, and O as identified in the EDS analysis conducted at point 5), which was evidence of the formation of reaction products as calcium silicate hydrates. For that reason, the enhancement in the mechanical properties of the stabilized MTZP was limited to the available Ca.

On the other hand, when a material was stabilized with Portland cement, a different series of reactions arise. Initially, when the water comes in contact with cement occurs the hydration, followed by the dissolution; release of Ca^2+^, Al^+3^, and SiO^−^ ions; formation of hydrated compounds as C-S–H and C-A-H; liberation of excess Ca(OH)_2_; and hardening of the mixture (Firoozi et al. 2017; Ferrazzo et al. 2023). Then the reactions accelerate, leading to increased strength and the growth of hydration products. Subsequently, the rate of reaction product growth decelerates, initiating the construction of the microstructure which remains in formation over time (Mindess et al. 2003). The FT-IR analysis (Fig. 3) illustrates the dissolution of specific clinker phases in Portland cement. A closer examination of the spectra revealed a stretching band at 927 cm^−1^ which was attributed to alite (tricalcium silicate), and at 873 and 845 cm^−1^ to belite (dicalcium silicate) (Yousuf et al. 1993; Domínguez et al. 2008; Taddei et al. 2009; Saedi et al. 2023). As the stretching vibrations were limited to the stabilizer and were not found in the PC-MTZP and QL-PC-MTZP mixtures, it suggests that they were consumed during the cement hydration process. Moreover, the stretching vibration at 3640 cm^−1^ attributed to the O–H bonds of portlandite (Zaki et al. 2006; Domínguez et al. 2008; García Lodeiro et al. 2009; Taddei et al. 2009; Jose et al. 2020) presented a lower intensity in Portland cement when compared with quicklime. This aspect clarifies the lower efficacy of solely using Portland cement for the treatment of sulfates. Due to the reduced amount of portlandite in PC, there was limited free calcium available to combine with sulfate to form calcium sulfate. Consequently, the levels of sulfates in the treatment with only Portland cement were higher than in the mixture containing solely quicklime, leading to the deterioration of the cement matrix and reduced strength. The observation of the microstructural aspects (Fig. 2c) revealed the presence of rod-shaped particles associated with the detection of Ca, S, and O, in the EDS analysis (point 2), which was evidence of the sulfate (Seewoo et al. 2004; Chen et al. 2021). It was evolved by a spongy morphology, composed of Ca, Si, and O as shown in EDS analysis (point 3), that was attributed to the reaction products as calcium silicate hydrates. However, in contrast to the QL-MTZP, the PC-MTZP mixture exhibited a smaller amount of these spongy morphologies surrounding the sulfate particles and a structure with a greater level of porosity. It was consistent with the mechanical response, where a lower strength and stiffness were observed in the PC-MTZP mixture.

The addition of both QL and PC to the MTZP produced a minimal enhancement in the mechanical response and a slight minor deviation in sulfate leaching. As discussed previously, the stabilization of the MTZP was greatly dependent on the available Ca in the system. Since the inclusion of PC does not provide a great increase in calcium in the system (the stretching vibration at 3640 cm^−1^ presented a reduced intensity), the results were similar to the treatment solely with QL. Although the incorporation of PC did not contribute to a significative enhancement in mechanical and environmental performance, it did lead to a decrease in the leaching of heavy metals as previously stated. The advantage of using both stabilizers was associated with reducing the leaching of sulfate through the action of QL and the encapsulation of the heavy metals by the PC. Despite not meeting all the regulatory thresholds, the application of both stabilizers effectively mitigates the risks associated with the disposal of the MTZP. Therefore, this treatment, when combined with strategies that diminish the contact of the treated material and water, offers a reduced potential for environmental contamination.

The combined microstructural, chemical, and mechanical analyses demonstrated that both quicklime and Portland cement promoted important changes in the MTZP material, though with distinct roles. Quicklime effectively contributed to sulfate immobilization through gypsum precipitation and supported the formation of cementitious products via pozzolanic reactions, improving strength at early ages. Portland cement, in turn, enhanced heavy metal stabilization by encapsulating contaminants within the cementitious matrix and promoting the formation of C-S–H and C-A-H gels. Despite the formation of reaction products and improvements in mechanical performance, the mechanical gains plateaued early, and overall strength and stiffness were still limited by calcium availability, especially when quicklime and cement were combined. While the dual treatment (QL and PC) offered advantages by simultaneously reducing sulfate and heavy metal leaching, the improvements in mechanical and environmental performance were modest, and some regulatory limits were not fully achieved. Nevertheless, this combined stabilization strategy represents a promising approach to mitigating environmental risks associated with MTZP disposal, especially when complemented by additional barriers to limit water contact.

Finally, it is important to mention that these findings should be interpreted within the context of certain limitations. The presented results apply to the early curing period, and long-term testing should be considered in future work to assess potential effects like carbonation, cracking, and wetting–drying. The stabilization efficacy reported herein is specific to the sulfate-resistant cement and the MTZP from silicate ore tested. The application of alternative cement types requires further validation studies to assess chemical compatibility, particularly regarding the potential risk of ettringite formation and the generation of brittle matrices associated with the presence of aluminate in the binders.

## Conclusions

This study aimed to evaluate the environmental, microstructural, and mechanical properties of metallurgical tailings from the zinc process from silicated ore stabilized with quicklime and blast furnace slag Portland cement (type III RS) to reduce the potential contamination, and some aspects were highlighted:

- Portland cement demonstrated high effectiveness in the stabilization of heavy metals, due to the encapsulation of the contaminants in the C-S–H gel.
- Quicklime stabilized the sulfates due to the common ion effect and the gypsum precipitation. Complementary, quicklime has a more pronounced effect on mechanical improvement in comparison with Portland cement.
- The combination of quicklime and Portland cement produced a synergistic effect. The combination of these binders provides a balanced enhancement of both environmental and mechanical stability.
- The presented results apply to the early curing period, and long-term testing should be considered in future work.

## Data Availability

Some or all data, models, or code that support the findings of this study are available from the corresponding author upon reasonable request.

## References

[CR1] Ahmari S, Zhang L (2013) Utilization of cement kiln dust (CKD) to enhance mine tailings-based geopolymer bricks. Constr Build Mater 40:1002–1011. 10.1016/j.conbuildmat.2012.11.069

[CR2] Akhter H, Butler LG, Branz S, Cartledge FK, Tittlebaum ME (1990) Immobilization of As, Cd, Cr and PB-containing soils by using cement or pozzolanic fixing agents. J Hazard Mater 24:145–155. 10.1016/0304-3894(90)87006-4

[CR3] Aldaood A, Bouasker M, Al-Mukhtar M (2021) Mechanical behavior of gypseous soil treated with lime. Geotech Geol Eng 39:719–733. 10.1007/s10706-020-01517-w

[CR4] Aldaood A, Bouasker M, Al-Mukhtar M (2014a) Geotechnical properties of lime-treated gypseous soils. Appl Clay Sci 88:39–48. 10.1016/j.clay.2013.12.015

[CR5] Aldaood A, Bouasker M, Al-Mukhtar M (2014b) Impact of wetting–drying cycles on the microstructure and mechanical properties of lime-stabilized gypseous soils. Eng Geol 174:11–21. 10.1016/j.enggeo.2014.03.002

[CR6] Aldaood A, Bouasker M, Al-Mukhtar M (2014c) Impact of freeze–thaw cycles on mechanical behaviour of lime stabilized gypseous soils. Cold Reg Sci Technol 99:38–45. 10.1016/j.coldregions.2013.12.003

[CR7] APHA, AWWA, WEF (2017) Standard methods for the examination of water and waste water. Washington, DC, USA.

[CR8] ABNT NBR 10004 (2004a) Solid waste – Classification. 1–71 (in Portuguese)

[CR9] ABNT NBR 10005 (2004b) Procedure for obtaining leached extract from solid waste. 1–16 (in Portuguese)

[CR10] ABNT NBR 10006 (2004c) Procedure for obtaining solubilized extract from solid waste. 1–3 (in Portuguese)

[CR11] ASTM D2487 (2017) Standard Practice for Classification of Soils for Engineering Purposes (Unified Soil Classification System). 1–10

[CR12] ASTM D2216 (2019) Standard Test Method for Laboratory Determination of Water (Moisture) Content of Soil and Rock by Mass. 1–7

[CR13] ASTM D2166 (2016) Standard Test Method for Unconfined Compressive Strength of Cohesive Soil. 1–7

[CR14] Astrup T, Dijkstra JJ, Comans RNJ, der Van Sloot HA, Christensen TH (2006) Geochemical modeling of leaching from MSWI air-pollution-control residues. Environ Sci Technol 40:3551–3557. 10.1021/es052250r 16786693 10.1021/es052250r

[CR15] Barjoveanu G, De Gisi S, Casale R, Todaro F, Notarnicola M, Teodosiu C (2018) A life cycle assessment study on the stabilization/solidification treatment processes for contaminated marine sediments. J Clean Prod 201:391–402. 10.1016/j.jclepro.2018.08.053

[CR16] Benatti CT, Tavares CRG, Lenzi E (2009) Sulfate removal from waste chemicals by precipitation. J Environ Manage 90:504–511. 10.1016/j.jenvman.2007.12.006 18222593 10.1016/j.jenvman.2007.12.006

[CR17] Bergado DT, Anderson LR, Miura N, Balasubramaniam AS (1996) Soft ground improvement in lowland and other environments. ASCE Press, New York

[CR18] Bulatović V, Melešev M, Radeka M, Radonjanin V, Lukić I (2017) Evaluation of sulfate resistance of concrete with recycled and natural aggregates. Constr Build Mater 152:614–631. 10.1016/j.conbuildmat.2017.06.161

[CR19] Bull AJ, Fall M (2020) Curing temperature dependency of the release of arsenic from cemented paste backfill made with Portland cement. J Environ Manage 269:1–13. 10.1016/j.jenvman.2020.110772 10.1016/j.jenvman.2020.110772 32560993

[CR20] Bye G (2011) Portland Cement. ICE Publishing, London

[CR21] Cai Y, Xiang Y, Zhou M, Wang H, Hou H (2025) Solidification/stabilization of copper tailings by industrial solid waste based cementing materials: apatite synergy, heavy metal toxicity and performance. Ceram Int. 10.1016/j.ceramint.2025.04.292

[CR22] Calgaro L, Contessi S, Bonetto A, Badetti E, Ferrari G, Artioli G, Marcomini A (2021) Calcium aluminate cement as an alternative to ordinary Portland cement for the remediation of heavy metals contaminated soil: mechanisms and performance. J Soils Sediments 21:1755–1768. 10.1007/s11368-020-02859-x

[CR23] Cartledge FK, Butler LG, Chalasani D, Eaton H, Frei F, Herrera E, Tittlebaum ME, Yang SL (1990) Immobilization mechanisms in solidification/stabilization of cadmium and lead salts using portland cement fixing agents. Environ Sci Technol 24:867–873. 10.1021/es00076a012

[CR24] Cetin B, Aydilek AH (2013) pH and fly ash type effect on trace metal leaching from embankment soils. Resour Conserv Recycl 80:107–117. 10.1016/j.resconrec.2013.09.006

[CR25] Chen Q, Luo K, Wang Y, Li X, Zhang Q, Liu Y (2022) In-situ stabilization/solidification of lead/zinc mine tailings by cemented paste backfill modified with low-carbon bentonite alternative. J Mater Res Technol 17:1200–1210. 10.1016/j.jmrt.2022.01.099

[CR26] Chen Q, Tao Y, Feng Y, Zhang Q, Liu Y (2021) Utilization of modified copper slag activated by Na2SO4 and CaO for unclassified lead/zinc mine tailings based cemented paste backfill. J Environ Manage. 10.1016/j.jenvman.2021.112608 10.1016/j.jenvman.2021.112608 33901826

[CR27] Chen QY, Tyrer M, Hills CD, Yang XM, Carey P (2009) Immobilisation of heavy metal in cement-based solidification/stabilisation: a review. Waste Manag 29:390–403. 10.1016/j.wasman.2008.01.019 18367391 10.1016/j.wasman.2008.01.019

[CR28] Chen W, Peng R, Straub C, Yuan B (2020) Promoting the performance of one-part alkali-activated slag using fine lead-zinc mine tailings. Constr Build Mater 236:117745. 10.1016/j.conbuildmat.2019.117745

[CR29] CONAMA 420 (2009) Provides criteria and guiding values ​​for soil quality regarding the presence of chemical substances and establishes guidelines for the environmental management of areas contaminated by these substances as a result of human activities. 1–16 (in Portuguese)

[CR30] Consoli NC, Bittar Marin EJ, Quiñónez Samaniego RA, Scheuermann Filho HC, Miranda T, Cristelo N (2019) Effect of mellowing and coal fly ash addition on behavior of sulfate-rich dispersive clay after lime stabilization. J Mater Civ Eng 31:1–10. 10.1061/(ASCE)MT.1943-5533.0002699

[CR31] Consoli NC, Cruz RC, Floss MF, Festugato L (2010) Parameters controlling tensile and compressive strength of artificially cemented sand. J Geotech Geoenviron Eng 136:759–763. 10.1061/(ASCE)GT.1943-5606.0000278

[CR32] Criado M, Fernández-Jiménez A, Palomo A (2007) Alkali activation of fly ash: effect of the SiO_2_/Na_2_O ratio. Microporous Mesoporous Mater 106:180–191. 10.1016/j.micromeso.2007.02.055

[CR33] Daniel DE, Wu Y-K (1993) Compacted clay liners and covers for arid sites. J Geotech Eng 119:223–237. 10.1061/(ASCE)0733-9410(1993)119:2(223)

[CR34] Deng L, Zhang Y, Chen F, Cao S, You S, Liu Y, Zhang Y (2013) Reactive crystallization of calcium sulfate dihydrate from acidic wastewater and lime. Chin J Chem Eng 21:1303–1312. 10.1016/S1004-9541(13)60626-6

[CR35] Desogus P, Manca PP, Orrù G, Zucca A (2013) Stabilization–solidification treatment of mine tailings using Portland cement, potassium dihydrogen phosphate and ferric chloride hexahydrate. Miner Eng 45:47–54. 10.1016/j.mineng.2013.01.003

[CR36] Do Carmo FF, Kamino LHY, Junior RT, De Campos IC, Do Carmo FF, Silvino G, De Castro KJSX, Mauro ML, Rodrigues NUA, Miranda MPS, Pinto CEF (2017) Fundão tailings dam failures: the environment tragedy of the largest technological disaster of Brazilian mining in global context. Perspect Ecol Conserv 15:145–151. 10.1016/j.pecon.2017.06.002

[CR37] Domínguez MI, Carpena J, Borschnek D, Centeno MA, Odriozola JA, Rose J (2008) Apatite and Portland/apatite composite cements obtained using a hydrothermal method for retaining heavy metals. J Hazard Mater 150:99–108. 10.1016/j.jhazmat.2007.04.091 17532119 10.1016/j.jhazmat.2007.04.091

[CR38] Doye I (2004) Neutralisation of acid mine drainage with alkaline industrial residues: laboratory investigation using batch-leaching tests. Fuel Energy Abstr 45:58. 10.1016/S0140-6701(04)91689-X

[CR39] Erdem M, Özverdi A (2011) Environmental risk assessment and stabilization/solidification of zinc extraction residue: II. stabilization/solidification. Hydrometallurgy 105:270–276. 10.1016/j.hydromet.2010.10.014

[CR40] Estep PA, Kovach JJ, Karr C (1968) Quantitative infrared multicomponent determination of minerals occurring in coal. Anal Chem 40:358–363. 10.1021/ac60258a006

[CR41] Fei J-C, Min X-B, Wang Z-X, Pang Z-H, Liang Y-J, Ke Y (2017) Health and ecological risk assessment of heavy metals pollution in an antimony mining region: a case study from South China. Environ Sci Pollut Res 24:27573–27586. 10.1007/s11356-017-0310-x 10.1007/s11356-017-0310-x 28980103

[CR42] Ferrazzo ST, Tonini de Araújo M, Bruschi GJ, Korf EP, Levandoski WMK, Pereira dos Santos C, Consoli NC (2023) Metal encapsulation of waste foundry sand stabilized with alkali-activated binder: batch and column leaching tests. J Environ Manage 348:119287. 10.1016/j.jenvman.2023.119287 37852081 10.1016/j.jenvman.2023.119287

[CR43] Firoozi AA, Guney Olgun C, Firoozi AA, Baghini MS (2017) Fundamentals of soil stabilization. Int J Geo-Eng 8:26. 10.1186/s40703-017-0064-9

[CR44] Fischer WR (1975) The formation of hematite from amorphous iron(III)hydroxide. Clays Clay Miner 23:33–37. 10.1346/CCMN.1975.0230105

[CR45] García Lodeiro I, Macphee DE, Palomo A, Fernández-Jiménez A (2009) Effect of alkalis on fresh C–S–H gels. FTIR analysis. Cem Concr Res 39:147–153. 10.1016/j.cemconres.2009.01.003

[CR46] García M, Chimenos J, Fernández A, Miralles L, Segarra M, Espiell F (2004) Low-grade MgO used to stabilize heavy metals in highly contaminated soils. Chemosphere 56:481–491. 10.1016/j.chemosphere.2004.04.005 15212914 10.1016/j.chemosphere.2004.04.005

[CR47] Ghigliazza R, Lodi A, Rovatti M (2000) Kinetic and process considerations on biological reduction of soluble and scarcely soluble sulfates. Resour Conserv Recycl 29:181–194. 10.1016/S0921-3449(99)00055-5

[CR48] Ghosh I, Guha S, Balasubramaniam R, Kumar AVR (2011) Leaching of metals from fresh and sintered red mud. J Hazard Mater 185:662–668. 10.1016/j.jhazmat.2010.09.069 21035262 10.1016/j.jhazmat.2010.09.069

[CR49] Giergiczny Z, Król A (2008) Immobilization of heavy metals (Pb, Cu, Cr, Zn, Cd, Mn) in the mineral additions containing concrete composites. J Hazard Mater 160:247–255. 10.1016/j.jhazmat.2008.03.007 18423859 10.1016/j.jhazmat.2008.03.007

[CR50] Gougar MLD, Scheetz BE, Roy DM (1996) Ettringite and C-S-H portland cement phases for waste ion immobilization: a review. Waste Manag 16:295–303. 10.1016/S0956-053X(96)00072-4

[CR51] Guimarães D, Leão VA (2014) Fundamental aspects related to batch and fixed-bed sulfate sorption by the macroporous type 1 strong base ion exchange resin Purolite A500. J Environ Manage 145:106–112. 10.1016/j.jenvman.2014.06.006 25014887 10.1016/j.jenvman.2014.06.006

[CR52] Gupta C, Prasad A (2018) Variables controlling strength of lime stabilized jarosite waste. Int J Geo-Eng 9:6. 10.1186/s40703-018-0074-2

[CR53] Han L, Xu Z, Shu J, Yu Y, Ning L, Gao S, Xu J, Li C (2023) Mn release behaviors from electrolytic manganese residue-based slow-release fertilizer using acid/alkali-activated geopolymers as binders: a comparative study. J Clean Prod 384:135497. 10.1016/j.jclepro.2022.135497

[CR54] Hofmeister H, Huisken F, Kohn B, Alexandrescu R, Cojocaru S, Crunteanu A, Morjan I, Diamandescu L (2001) Filamentary iron nanostructures from laser-induced pyrolysis of iron pentacarbonyl and ethylene mixtures. Appl Phys A Mater Sci Process 72:7–11. 10.1007/s003390000599

[CR55] Hossein M (2000) Rule of ettringite formation in the stabilization/solidification of sulphide-bearing mine waste. Thesis (Doctor of Philosophy) - Department of Mining and Metallurgical Engineering, McGill Universit

[CR56] Hu P, Zhang Y, Liu T, Huang J, Yuan Y, Zheng Q (2017) Highly selective separation of vanadium over iron from stone coal by oxalic acid leaching. J Ind Eng Chem 45:241–247. 10.1016/j.jiec.2016.09.029

[CR57] Huang G, Rao X, Shao X, Gu Q, Wang Z, Li P, Huang J (2023) Distribution of heavy metals influenced by pumped storage hydropower in abandoned mines: leaching test and modelling simulation. J Environ Manage 326:116836. 10.1016/j.jenvman.2022.116836 36435130 10.1016/j.jenvman.2022.116836

[CR58] Ineich T, Degreve C, Karamoutsos S, du Plessis C (2017) Utilization efficiency of lime consumption during magnesium sulfate precipitation. Hydrometallurgy 173:241–249. 10.1016/j.hydromet.2017.09.001

[CR59] Ingles OG, Metcalf JB (1973) Soil stabilization - principles and practice. John Wiley & Sons, Inc, New York

[CR60] Ji J, Ge Y, Balsam W, Damuth JE, Chen J (2009) Rapid identification of dolomite using a Fourier Transform infrared spectrophotometer (FTIR): a fast method for identifying Heinrich events in IODP Site U1308. Mar Geol 258:60–68. 10.1016/j.margeo.2008.11.007

[CR61] Jose A, Nivitha MR, Krishnan JM, Robinson RG (2020) Characterization of cement stabilized pond ash using FTIR spectroscopy. Constr Build Mater 263:120136. 10.1016/j.conbuildmat.2020.120136

[CR62] Kamel AMA, Marie HAH, Mahmoud HA, Ali MF (2015) Mineralogical characterization of Islamic stucco: minaret of Shams El-Deen El-Wasty, Bulaq, Egypt. Constr Build Mater 101:692–701. 10.1016/j.conbuildmat.2015.10.059

[CR63] Khamseh B, Shourijeh PT, Binesh SM (2025) Strength and deformation characteristics of cement-stabilized Fe-rich fine iron ore tailings. Constr Build Mater 463:140101. 10.1016/j.conbuildmat.2025.140101

[CR64] Khan HA, Castel A, Khan MSH, Mahmood AH (2019) Durability of calcium aluminate and sulphate resistant Portland cement based mortars in aggressive sewer environment and sulphuric acid. Cem Concr Res 124:105852. 10.1016/j.cemconres.2019.105852

[CR65] Khoeurn K, Sakaguchi A, Tomiyama S, Igarashi T (2019) Long-term acid generation and heavy metal leaching from the tailings of Shimokawa mine, Hokkaido, Japan: column study under natural condition. J Geochem Explor 201:1–12. 10.1016/j.gexplo.2019.03.003

[CR66] Król M, Minkiewicz J, Mozgawa W (2016) IR spectroscopy studies of zeolites in geopolymeric materials derived from kaolinite. J Mol Struct 1126:200–206. 10.1016/j.molstruc.2016.02.027

[CR67] Ladd RS (1979) Preparing test specimens using undercompaction. Int J Rock Mech Min Sci Geomech Abstr 16:50. 10.1016/0148-9062(79)90502-3

[CR68] Lane MD, Christensen PR (1997) Thermal infrared emission spectroscopy of anhydrous carbonates. J Geophys Res Planets 102:25581–25592. 10.1029/97JE02046

[CR69] Lin Y, Ma J, Zhang Z, Zhu Y, Hou H, Zhao L, Sun Z, Xue W, Shi H (2018) Linkage between human population and trace elements in soils of the Pearl River Delta: implications for source identification and risk assessment. Sci Total Environ 610:944–950. 10.1016/j.scitotenv.2017.08.147 28830054 10.1016/j.scitotenv.2017.08.147

[CR70] Liu B, Peng T, Sun H (2017) Leaching behavior of U, Mn, Sr, and Pb from different particle-size fractions of uranium mill tailings. Environ Sci Pollut Res 24:15804–15815. 10.1007/s11356-017-8921-9 10.1007/s11356-017-8921-9 28534266

[CR71] Luo Z, Tang C, Hao Y, Wang Z, Yang G, Wang Y, Mu Y (2022) Solidification/stabilization of heavy metals and its efficiency in lead–zinc tailings using different chemical agents. Environ Technol 43:1613–1623. 10.1080/09593330.2020.1845817 33135954 10.1080/09593330.2020.1845817

[CR72] Ma J, Wang Q, Jiao H, Li Z, Li G, Xu P, Zou S, Yang L, Liu X (2024) Solidification/stabilization and leaching behavior of heavy metals in low-binder cemented tailings backfill. Case Stud Constr Mater 21:e03934. 10.1016/j.cscm.2024.e03934

[CR73] Mafessoli M, Marques SFV, Scheuermann Filho HC, Consoli NC (2023) Response of artificially cemented iron ore tailings for dry stacking disposal over a wide range of stresses. Indian Geotech J 53:904–915. 10.1007/s40098-023-00711-w

[CR74] Mahedi M, Cetin B, Dayioglu AY (2019) Leaching behavior of aluminum, copper, iron and zinc from cement activated fly ash and slag stabilized soils. Waste Manag 95:334–355. 10.1016/j.wasman.2019.06.018 31351620 10.1016/j.wasman.2019.06.018

[CR75] Malviya R, Chaudhary R (2006) Leaching behavior and immobilization of heavy metals in solidified/stabilized products. J Hazard Mater 137:207–217. 10.1016/j.jhazmat.2006.01.056 16504383 10.1016/j.jhazmat.2006.01.056

[CR76] McGregor R, Blowes D, Jambor J, Robertson W (1998) The solid-phase controls on the mobility of heavy metals at the Copper Cliff tailings area, Sudbury, Ontario, Canada. J Contam Hydrol 33:247–271. 10.1016/S0169-7722(98)00060-6

[CR77] Mechri ML, Chihi S, Mahdadi N, Beddiaf S (2017) Diagnosis of the heating effect on the electrical resistivity of Ouargla (Algeria) dunes sand using XRD patterns and FTIR spectra. J African Earth Sci 125:18–26. 10.1016/j.jafrearsci.2016.10.007

[CR78] Mindess S, Young JF, Darwin D (2003) Concrete. 657

[CR79] Mitchell JK (1981) Soil improvement — state-of-the-art report. In: Proceedings of the 10th International Conference on Soil Mechanics and Foundation Engineering. pp 509–565

[CR80] Mitchell JK, Soga K (2005) Fundamentals of soil behavior. John Wiley & Sons, Inc, New York, US

[CR81] Moukannaa S, Loutou M, Benzaazoua M, Vitola L, Alami J, Rakkou R (2018) Recycling of phosphate mine tailings for the production of geopolymers. J Clean Prod 185:891–903. 10.1016/j.jclepro.2018.03.094

[CR82] Mymrin V, Vazquez Vaamonde A (1999) New construction materials from Spanish jarosite processing wastes. Miner Eng 12:1399–1402. 10.1016/S0892-6875(99)00126-0

[CR83] Nariyan E, Wolkersdorfer C, Sillanpää M (2018) Sulfate removal from acid mine water from the deepest active European mine by precipitation and various electrocoagulation configurations. J Environ Manage 227:162–171. 10.1016/j.jenvman.2018.08.095 30176436 10.1016/j.jenvman.2018.08.095

[CR84] Nehdi M, Tariq A (2007) Stabilization of sulphidic mine tailings for prevention of metal release and acid drainage using cementitious materials: a review. J Environ Eng Sci 6:423–436. 10.1139/s06-060

[CR85] Nicholson PG (2015) Soil improvement and ground modification methods. Butterworth-Heinemann, Waltham, MA

[CR86] Othmani MA, Souissi F, Benzaazoua M, Bouzahzah H, Bussiere B, Mansouri A (2013) The geochemical behaviour of mine tailings from the Touiref Pb–Zn district in Tunisia in weathering cells leaching tests. Mine Water Environ 32:28–41. 10.1007/s10230-012-0210-8

[CR87] Özkök E, Davis AP, Aydilek AH (2013) Leaching of As, Cr, and Cu from high-carbon fly ash–soil mixtures. J Environ Eng 139:1397–1408. 10.1061/(ASCE)EE.1943-7870.0000751

[CR88] Pacheco-Torgal F, Labrincha JA, Leonelli C, Palomo A, Chindaprasirt P (2015) Handbook of Alkali-activated Cements, Mortars and Concretes. Woodhead, Cambridge

[CR89] Park C-K (2000) Hydration and solidification of hazardous wastes containing heavy metals using modified cementitious materials. Cem Concr Res 30:429–435. 10.1016/S0008-8846(99)00272-0

[CR90] Peng J, Zhang S, Han Y, Bate B, Ke H, Chen Y (2022) Soil heavy metal pollution of industrial legacies in China and health risk assessment. Sci Total Environ 816:151632. 10.1016/j.scitotenv.2021.151632 34780826 10.1016/j.scitotenv.2021.151632

[CR91] Pereira dos Santos C, Bruschi GJ, Tonatto Ferrazzo S, Kubiaki Levandoski WM, Pavan Korf E, Consoli NC (2024) Leaching behavior of alkali-activated gold tailings over wetting–drying cycles. Indian Geotech J. 10.1007/s40098-024-00886-w

[CR92] Piciullo L, Storrøsten EB, Liu Z, Nadim F, Lacasse S (2022) A new look at the statistics of tailings dam failures. Eng Geol 303:14. 10.1016/j.enggeo.2022.106657

[CR93] Prusinski JR, Bhattacharja S (1999) Effectiveness of Portland cement and lime in stabilizing clay soils. Transp Res Rec J Transp Res Board 1652:215–227. 10.3141/1652-28

[CR94] Rai S, Bahadure S, Chaddha MJ, Agnihotri A (2020) Disposal practices and utilization of red mud (bauxite residue): a review in Indian context and abroad. J Sustain Metall 6:1–8. 10.1007/s40831-019-00247-5

[CR95] Reig F (2002) FTIR quantitative analysis of calcium carbonate (calcite) and silica (quartz) mixtures using the constant ratio method. Application to geological samples. Talanta 58:811–821. 10.1016/S0039-9140(02)00372-7 18968811 10.1016/s0039-9140(02)00372-7

[CR96] Ribeiro L, Rios S, Arroyo M, Mánica M (2026) A parametric study on the stability of tailings storage facilities with cemented berms. Soils Rocks 49:e2026009424. 10.28927/SR.2026.009424

[CR97] Rogers CDF, Glendinning S (2000) Lime requirement for stabilization. Transp Res Rec J Transp Res Board 1721:9–18. 10.3141/1721-02

[CR98] Romero FM, Armienta MA, González-Hernández G (2007) Solid-phase control on the mobility of potentially toxic elements in an abandoned lead/zinc mine tailings impoundment, Taxco, Mexico. Appl Geochem 22:109–127. 10.1016/j.apgeochem.2006.07.017

[CR99] Roy A, Bhattacharya J (2015) Nanotechnology in industrial wastewater treatment. IWA Publishing, London

[CR100] Saedi A, Jamshidi-Zanjani A, Mohseni M, Khodadadi Darban A, Nejati H (2023) Mechanical activation of lead–zinc mine tailings as a substitution for cement in concrete construction. Constr Build Mater 364:129973. 10.1016/j.conbuildmat.2022.129973

[CR101] Sáez del Bosque IF, Martínez-Ramírez S, Blanco-Varela MT (2014) FTIR study of the effect of temperature and nanosilica on the nano structure of C-S–H gel formed by hydrating tricalcium silicate. Constr Build Mater 52:314–323. 10.1016/j.conbuildmat.2013.10.056

[CR102] Sarma LP, Prasad PSR, Ravikumar N (1998) Raman spectroscopic study of phase transitions in natural gypsum. J Raman Spectrosc 29:851–856. 10.1002/(SICI)1097-4555(199809)29:9<851::AID-JRS313>3.0.CO;2-S

[CR103] Seewoo S, Van Hille R, Lewis A (2004) Aspects of gypsum precipitation in scaling waters. Hydrometallurgy 75:135–146. 10.1016/j.hydromet.2004.07.003

[CR104] Sethurajan M, Huguenot D, Lens PNL, Horn HA, Figueiredo LHA, Van Hullebusch ED (2016) Fractionation and leachability of heavy metals from aged and recent Zn metallurgical leach residues from the Três Marias zinc plant (Minas Gerais, Brazil). Environ Sci Pollut Res 23:7504–7516. 10.1007/s11356-015-6014-1 10.1007/s11356-015-6014-1 26728285

[CR105] Sheen RT, Kahler HL, Ross EM, Betz HA, Betz LD (1935) Turbidimetric determination of sulfate in water. Ind Eng Chem Anal Ed 7:262–265. 10.1021/ac50096a022

[CR106] Shnorhokian S (1996) Immobilization of heavy metals in lime-fly ash cementitious binders. Thesis (Master of Science) - Department of Mining and Metallurgical Engineering, McGill University

[CR107] Sidhu PS (1988) Transformation of trace element-substituted maghemite to hematite. Clays Clay Miner 36:31–38. 10.1346/CCMN.1988.0360105

[CR108] Silva A, Varesche M, Foresti E, Zaiat M (2002) Sulphate removal from industrial wastewater using a packed-bed anaerobic reactor. Process Biochem 37:927–935. 10.1016/S0032-9592(01)00297-7

[CR109] Silva Rotta LH, Alcântara E, Park E, Negri RG, Lin YN, Bernardo N, Mendes TSG, Souza Filho CR (2020) The 2019 brumadinho tailings dam collapse: possible cause and impacts of the worst human and environmental disaster in Brazil. Int J Appl Earth Obs Geoinf 90:102119. 10.1016/j.jag.2020.102119

[CR110] Spence RD (1993) Chemistry and microstructure of solidified waste forms. Lewis Publishers, Boca Raton

[CR111] Spence RD, Shi C (2005) Stabilization and solidification of hazardous, radioactive, and mixed wastes. CRC Press, Boca Raton

[CR112] Taddei P, Tinti A, Gandolfi MG, Rossi PL, Prati C (2009) Vibrational study on the bioactivity of Portland cement-based materials for endodontic use. J Mol Struct 924:548–554. 10.1016/j.molstruc.2008.11.002

[CR113] Taylor HFW (1997) Cement chemistry. Thomas Telford Publishing, London

[CR114] Thompson F, de Oliveira BC, Cordeiro MC, Masi BP, Rangel TP, Paz P, Freitas T, Lopes G, Silva BS, Cabral AS, Soares M, Lacerda D, Dos Santos Vergilio C, Lopes-Ferreira M, Lima C, Thompson C, De Rezende CE (2020) Severe impacts of the Brumadinho dam failure (Minas Gerais, Brazil) on the water quality of the Paraopeba River. Sci Total Environ 705:1–6. 10.1016/j.scitotenv.2019.135914 10.1016/j.scitotenv.2019.135914 31838417

[CR115] Transportation Research Board (1987) State of the Art Report 5: lime stabilization – reactions properties, design and construction. State-of-the-Art Rep. No. 5 64

[CR116] U.S. Army Corps of Engineers (1994) Soil Stabilization for Pavements. 1–9

[CR117] USEPA (2009) National Primary Drinking Water Guidelines. 1–7

[CR118] Vempati RK (1990) Infrared vibrations of hematite formed from aqueous- and dry-thermal incubation of Si-containing ferrihydrite. Clays Clay Miner 38:294–298. 10.1346/CCMN.1990.0380308

[CR119] Viana da Fonseca A, Caetano I, Meneses B, Rios S (2024) Tailing storage facilities with cemented berms for sustainable production of raw materials. In: Proceedings of the 5th International Conference on Geotechnics for Sustainable Infrastructure Development - GEOTEC HANOI 2023. ISSMGE, Hanoi, Vietnam, pp 1099–1111

[CR120] Vollpracht A, Brameshuber W (2016) Binding and leaching of trace elements in Portland cement pastes. Cem Concr Res 79:76–92. 10.1016/j.cemconres.2015.08.002

[CR121] VROM (2000) Dutch Target and Intervention Values. 1–51

[CR122] Wan Q, Rao F, Song S, Zhang Y (2019) Immobilization forms of ZnO in the solidification/stabilization (S/S) of a zinc mine tailing through geopolymerization. J Mater Res Technol 8:5728–5735. 10.1016/j.jmrt.2019.09.040

[CR123] Wang D, Wang Q, Xue J (2020) Reuse of hazardous electrolytic manganese residue: detailed leaching characterization and novel application as a cementitious material. Resour Conserv Recycl 154:104645. 10.1016/j.resconrec.2019.104645

[CR124] Wang F, Li W, Wang H, Hu Y, Cheng H (2024) The leaching behavior of heavy metal from contaminated mining soil: the effect of rainfall conditions and the impact on surrounding agricultural lands. Sci Total Environ 914:169877. 10.1016/j.scitotenv.2024.169877 38185143 10.1016/j.scitotenv.2024.169877

[CR125] Wang F, Long G, Zhou JL (2023) Deep insight into green remediation and hazard-free disposal of electrolytic manganese residue-based cementitious material. Sci Total Environ 894:165049. 10.1016/j.scitotenv.2023.165049 37355110 10.1016/j.scitotenv.2023.165049

[CR126] Wang H, Ju C, Zhou M, Chen J, Dong Y, Hou H (2022) Sustainable and efficient stabilization/solidification of Pb, Cr, and Cd in lead-zinc tailings by using highly reactive pozzolanic solid waste. J Environ Manage 306:114473. 10.1016/j.jenvman.2022.114473 35026710 10.1016/j.jenvman.2022.114473

[CR127] Yaseri S, Masoomi Verki V, Mahdikhani M (2019) Utilization of high volume cement kiln dust and rice husk ash in the production of sustainable geopolymer. J Clean Prod 230:592–602. 10.1016/j.jclepro.2019.05.056

[CR128] Ye M, Li G, Yan P, Ren J, Zheng L, Han D, Sun S, Huang S, Zhong Y (2017) Removal of metals from lead-zinc mine tailings using bioleaching and followed by sulfide precipitation. Chemosphere 185:1189–1196. 10.1016/j.chemosphere.2017.07.124 28772358 10.1016/j.chemosphere.2017.07.124

[CR129] Yousuf M, Mollah A, Hess TR, Tsai Y-N, Cocke DL (1993) An FTIR and XPS investigations of the effects of carbonation on the solidification/stabilization of cement based systems-Portland type V with zinc. Cem Concr Res 23:773–784. 10.1016/0008-8846(93)90031-4

[CR130] Yu P, Kirkpatrick RJ, Poe B, McMillan PF, Cong X (1999) Structure of calcium silicate hydrate (C-S-H): near-, mid-, and far-infrared spectroscopy. J Am Ceram Soc 82:742–748. 10.1111/j.1151-2916.1999.tb01826.x

[CR131] Zaki MI, Knözinger H, Tesche B, Mekhemer GAH (2006) Influence of phosphonation and phosphation on surface acid–base and morphological properties of CaO as investigated by in situ FTIR spectroscopy and electron microscopy. J Colloid Interface Sci 303:9–17. 10.1016/j.jcis.2006.07.011 16934283 10.1016/j.jcis.2006.07.011

[CR132] Zhang W, Zhao L, Yuan Z, Li D, Morrison L (2021) Assessment of the long-term leaching characteristics of cement-slag stabilized/solidified contaminated sediment. Chemosphere. 10.1016/j.chemosphere.2020.128926 10.1016/j.chemosphere.2020.128926 33243571

[CR133] Zhang Y, Jing Z, Kameda T, Yoshioka T (2016) Hydrothermal synthesis of hardened diatomite-based adsorbents with analcime formation for methylene blue adsorption. RSC Adv 6:26765–26774. 10.1039/C5RA18948A

[CR134] Zheng J, Guo L, Sun X, Li W, Jia Q (2018) Study on the strength development of cemented backfill body from lead-zinc mine tailings with sulphide. Adv Mater Sci Eng 2018:1–8. 10.1155/2018/7278014

